# Iodine content of six fish species, Norwegian dairy products and hen’s egg

**DOI:** 10.29219/fnr.v62.1291

**Published:** 2018-05-24

**Authors:** Ive Nerhus, Maria Wik Markhus, Bente M. Nilsen, Jannike Øyen, Amund Maage, Elisabeth Rasmussen Ødegård, Lisa Kolden Midtbø, Sylvia Frantzen, Tanja Kögel, Ingvild Eide Graff, Øyvind Lie, Lisbeth Dahl, Marian Kjellevold

**Affiliations:** Institute of Marine Research (IMR), Bergen, Norway

**Keywords:** iodine, dairy products, fish, hen’s egg, ICP-MS, food analysis, food composition table

## Abstract

Iodine is a trace element required for the production of thyroid hormones, essential for metabolism, growth and brain development, particularly in the first trimester of pregnancy. Milk and lean fish are the main dietary sources of iodine in the Norwegian diet. Thus, the aim of the present study was to provide updated analysed values of iodine concentration in six fish species, 27 selected Norwegian iodine-rich dairy foods and Norwegian hen’s eggs. The iodine concentrations in the wild fish species varied between 18 μg/100 g (Atlantic halibut) and 1,210 μg/100 g (pollack). The iodine concentration of cow milk varied between 12 and 19 μg/100 g and the iodine concentration of the eggs varied between 23 and 43 μg/100 g. The results in this study deviate somewhat from the current iodine concentrations in the Norwegian Food Composition Table. This deviation may have a large impact on the assessment of the iodine intake. Hence, updated knowledge about the variation in iodine level of fish, milk, dairy products and hen’s egg are of great importance when estimating the iodine intake in the population. These data will contribute substantially to future estimations of dietary iodine intake and will be made available for the public Norwegian Food Composition Table.

Iodine is an important trace element in human nutrition and is essential for thyroid hormone synthesis. Thyroid hormones are involved in many cellular activities essential for normal body function. The importance of assessing iodine intake of pregnant and lactating women has become increasingly clear because of emerging evidence from cohort studies showing that even mild to moderate iodine deficiency during pregnancy is associated with poorer cognitive function and school performance in children (1–4).

Iodine in sufficient concentrations occurs naturally in only a limited variety of foods, marine fish having the highest iodine concentrations in general (5, 6). Iodine concentrations vary between and within fish species, and also seasonally and geographically location, as fish absorb iodine both from the seawater and from their food (7). Even though their iodine content is considerably lower than that of fish, milk and dairy products are the iodine sources of greatest importance due to their common consumation in larger quantities in the Norwegian culture. The iodine concentration of milk depends on the supplementation concentratrion of iodine of cow feed, the amount of goitrogens in the rations, application of teat dipping containing iodine, iodine source, lactation stage, milk yield and milk processing (8). In addition, egg is a good dietary source of iodine (5, 9). To determine the iodine content of biological samples, inductively coupled plasma-mass spectrometry (ICP-MS) is the preferred method due to its precision.

Iodised salt programmes are the recommended method for providing sufficient iodine intake in a population (10). However, in countries with low availability of iodised salt, the intake of dietary iodine intake is of outmost importance. Neither household nor industrial iodisation of salt is mandatory in Norway. However, some brands have added iodine (5 mg/kg) in salt (6). Sufficient iodine intake is still challenging in population groups across the world, including countries in Europe (11, 12). In the Norwegian diet, milk and dairy products contribute with 55% and fish contributes with 20% of the dietary iodine intake (6). Iodine was included in the Norwegian Food Composition Table in 2014. Dietary iodine sources in the Nordic countries have several similarities; in Denmark, milk and mandatory fortification of salt used in the bakery industry and household salt are the main dietary iodine sources (11, 13). In Iceland, it is fish; in Sweden, iodised salt; and in Finland, milk (11). In the United Kingdom, which is also lacking an iodised salt programme, milk and dairy products contribute most to the dietary iodine intake (4). Knowledge about current iodine content in foods is expedient for several reasons. Since there are few dietary sources of iodine, it is important and feasible to map to what extent these food groups contribute. In addition, it is important to estimate iodine intake of population groups, which will provide indications on whether additional monitoring programmes or health care information for vulnerable groups are necessary. Thus, the main aim of the present study was to provide updated data on the iodine content of the most important iodine-rich food groups in the Norwegian diet, dairy products and lean fish. In addition, updated data on egg was provided. Since Norway is a major provider of fish to Europe, the data also have high relevance for calculations of European dietary iodine intake.

## Materials and methods

### Sampling of fish species

**Fig. 1:**
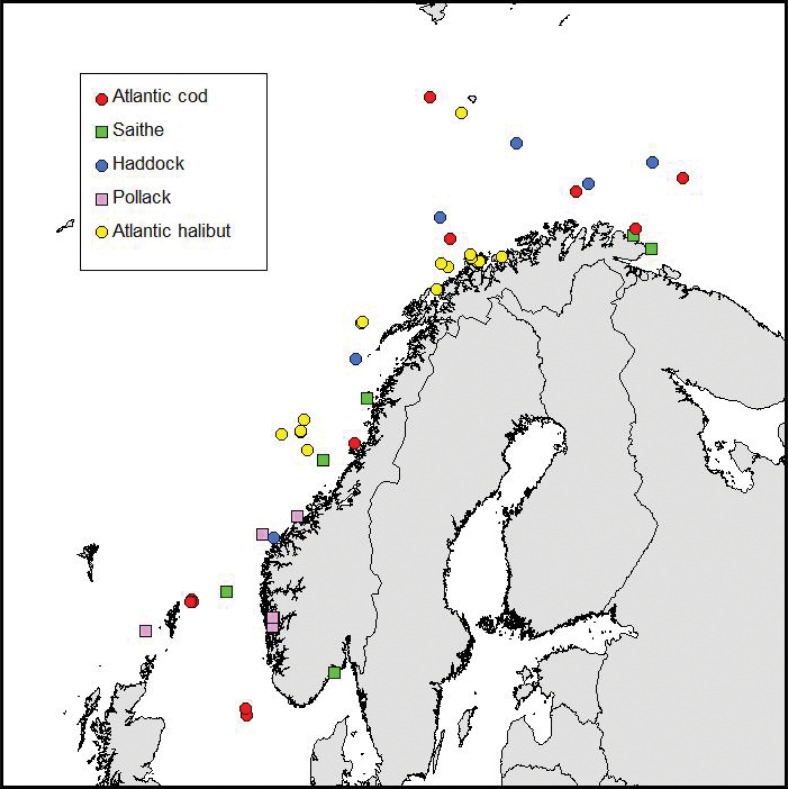
Sampling positions for Atlantic cod, saithe, haddock, pollack and Atlantic halibut. Cod, saithe and haddock, 10–11 fish per position; pollack, 6–11 fish per position; Atlantic halibut, 1–2 fish per position.

Five different species of wild fish were included in this study – Atlantic cod (*Gadus morhua*), saithe (*Pollachius virens*), haddock (*Melanogrammus aeglefinus*), pollack (*Pollachius pollachius*) and Atlantic halibut (*Hippoglossus hippoglossus*). Figure 1 illustrates sampling positions. Table 1 lists information regarding sampling period, number of positions and number of fish collected within the three major different sea areas – the Barents Sea, the Norwegian Sea and the North Sea.

**Table 1:** Overview of wild fish sampled for analysis of iodine content

Fish species and sampling area	Sampling period	Number of positions	Number of fish
*Atlantic cod* (*Gadus morhua*)		11	121
Barents Sea	January–March 2014 and February 2015	5	55
Norwegian Sea	October 2014	1	11
North Sea	August–September 2014	5	55
*Saithe* (*Pollachius virens*)		6	61
Barents Sea	July 2013 and June 2015	2	20
Norwegian Sea	March–April 2014	2	20
North Sea and Skagerrak	March and May 2014	2	21
*Haddock* (*Melanogrammus aeglefinus*)		6	65
Barents Sea	January–March 2015	4	43
Norwegian Sea	February and May 2015	2	22
*Pollack* (*Pollachius pollachius*)		5	41
* *Norwegian Sea	April and August 2014	2	17
* *North Sea	June 2014	1	6
Fjords in western Norway	April, October and November 2014	2	18
*Atlantic halibut* (*Hippoglossus hippoglossus*)		19	20
Barents Sea	September–October 2014	9	10
Norwegian Sea	August–October 2014	10	10

The samples of wild fish analysed in this study were collected during several monitoring programmes for contaminants in wild fish in Norwegian sea areas (14–19) (Kögel, T. personal communication). The most recent samples collected for each species and area were selected, and most samples included were collected in 2014 and 2015. The sampling positions for each species were selected to obtain samples from a wide geographical area aiming to represent the normal commercial fishery catch areas. From each position, 10–11 individual fish of intermediate size were included in the study, excluding the smallest and largest fish in order to reduce the possible biological variation of the iodine concentration due to the fish size. However, if less than 10 fish were collected at a particular position, all fish were included into the study regardless of size. For Atlantic halibut, only one (or in one case two) fish were collected at each position. To obtain representative results for this species, 10 fish from the Barents Sea and 10 fish from the Norwegian Sea were included, selecting fish with weights between 11 and 40 kg, which is the most common commercial weight class for this species.

Furthermore, samples of 40 farmed Atlantic halibut were collected at three different fish farms in the western part of Norway in March, June and July 2014 and in April, August and September 2015. In addition to the fish samples taken in the field, 10 different products of canned tuna, from seven different brands, were purchased in various supermarkets in Bergen in November 2015.

### Preparation of fish

Samples of Atlantic cod, saithe, pollack and haddock were frozen as whole fish at −20°C before being shipped to the Institute of Marine Research, Bergen, Norway. For Atlantic halibut, length, weight and sex of each fish were recorded immediately after capture, and from each fish, a sample including the head and a 20–30 cm section anterior of the head was frozen and shipped to the laboratory. The samples of wild Atlantic halibut were thawed and a sample of about 200 g muscle tissue from the lean part of the fillet (B-cut) on the upper, ventral side of the fish anterior of the pectoral fin of each fish (20) was homogenised. The samples of cod, saithe, haddock and pollack were thawed, and the length, weight and sex of each fish was determined. The fish were filleted, skinned and about 200 g of muscle tissue from each individual fish was homogenised. The wet homogenised fish samples were freeze-dried, ground to a fine powder, homogenised again and kept dry pending analysis. Analyses of wild fish were performed on individual fish samples. The samples of farmed Atlantic halibut were thawed, filleted and skinned. Muscle samples from five fish collected at the same fish farm at the same time were pooled and homogenised. The pooled samples were freeze-dried, ground to a fine powder, homogenised again and kept dry pending analysis. For the canned tuna, any oil or water in the respective products was removed before the samples were homogenised and freeze-dried.

### Sampling of dairy products

Seven different types of cow milk, one type of soy milk, two different types of cream milk and 17 other dairy products were included (Table 2). All products were produced in Norway with the exception of soy milk and two types of cream cheese. During the last years, alternative ‘dairy’ products, such as soy and oat milk, have become popular. Therefore, soymilk with natural flavour was selected for analysis based on supermarket volume. Information regarding producer, country of production, batch number, best before date and place of sampling were registered. All products were purchased with three different batch numbers at supermarkets in Bergen, Norway and stored in a refrigerator (4°C) prior to homogenisation.

**Table 2:** Norwegian milk and dairy products sampled for analysis of iodine content

Dairy product	*n*	Description and fat content
*Milk*		
Low-fat milk, TINE	9	Cow milk, 1.2% fat
Skimmed milk, TINE	9	Cow milk, 0.1% fat
Organic low-fat milk, TINE	9	Cow milk, 1.2% fat
Organic low-fat milk, Røros Meieri	9	Cow milk, 1.2% fat
Low-fat milk, Q	9	Cow milk, 1.0% fat
Skimmed milk, Q	9	Cow milk, 0.5% fat
Chocolate-flavoured low-fat milk. Q	3	Cow milk, 1.2% fat
Soy milk, Alpro	3	Soy beans, 1.8% fat
*Probiotic milk with LGG*		
Biola with blueberry flavour, TINE	3	Cow milk, 0.1% fat
*Yoghurt*		
‘Go’morgen’ with flavour, TINE	3	Cow milk and contains müsli, 3% fat
Yoghurt with natural flavour, TINE	3	Cow milk, 3.4% fat
*White-coloured solid cheese*		
‘Norvegia’, TINE	3	Cow milk, 2% fat
‘Jarlsberg’, TINE	3	Cow milk, 27% fat
‘Norsk gulost’, Synnøve Finden	3	Cow milk, 26% fat
*White soft and cream cheeses*		
* *Brie, Arla Foods, HØNG	3	Ripened cheese of pasteurised cow milk, 34%
* *Camembert, TINE	3	Ripened cheese of pasteurised cow milk, 28%
Soft, cream cheese, ‘Snøfrisk’, TINE	3	Cream cheese of goat milk, 25% fat
Soft, cream cheese, ‘Philadelphia’, Mondalez	3	Cream cheese of cow milk, 23.5% fat
*Whey cheese*		
‘Gudbrandsdalsost’, TINE	3	Cow milk and goat milk, 29% fat
‘Fløtemysost’, TINE	3	Cow milk, 27% fat
‘Ekte geitost’, TINE	3	Goat milk, 27% fat
*Other dairy products*		
Cream milk, TINE	9	Cream milk, 38% fat
Cream milk, TINE	9	Cream milk, 20% fat
Crème fraiche, TINE	3	Curdled cream, 35% fat
Cottage cheese, TINE	3	Cheese product, 4.3% fat
Curd with natural flavour, TINE	3	Curd/Quark, 8.1% fat
Sour cream, TINE	3	Sour cream, 18% fat

### Preparation of dairy products

The homogenisation procedure of cow milk, chocolate-flavoured milk, cream milk, soy milk and probiotic milk (liquids) consisted of the following steps: a subsample of 200 mL from each of three different batch numbers were mixed in a glass bottle, resulting in one pooled sample of 600 mL. From this, two subsamples were frozen pending analysis (12.5 mL) and one for back up (50 mL). Three pooled samples were analysed at each sampling occasion. Furthermore, cow milk and cream milks were sampled at three different occasions of the year (Table 6).

The homogenisation procedure of yoghurts, sour cream, crème fraiche, cottage cheese, brie, camembert and solid cheeses consisted of the following steps: three items of each product were mixed and homogenised in a kitchen machine (Braun, lab.nr 1598), resulting in one pooled sample. Two sub-samples of 12.5 mL and 50 mL were collected, one for freeze-drying, pending analysis, and one for backup, respectively. All backups were stored in −80°C freezer.

A subsample of the dairy products (except probiotic milk, cow milk, chocolate-flavoured cow milk, soymilk and cream milks) was freeze-dried for minimum 24 h at −20°C or −80°C, (Labconco Freezone, 18 liter, model 775030).

### Sampling of hen’s egg

Eggs from three different Norwegian producers were purchased in shops in Bergen in April 2016 and in Bergen and Oslo April 2017. Based on information on their market share, the brand with the highest market share was selected. Each box of egg consisted of 6 or 12 eggs and in total 33 boxes were purchased (Table 8). Eggs purchased in 2016 were stored at −80°C until analysis and the sample from 2017 was stored fresh at 4°C until analysis.

### Preparation of hen’s eggs

Each pooled hen’s egg sample from 2016 consisted of 36 eggs from three different boxes (same brand), while each pooled hen’s egg sample from 2017 consisted of 18 eggs from three different boxes (same brand). The pooled egg samples were homogenised using a kitchen whisk.

### Iodine analysis and accuracy of the measurements

The iodine content was determined using inductive coupled plasma-mass spectrophotometry (ICP-MS). Tetra methyl ammonium hydroxide (TMAH) and water were added to the samples before extraction at 90°C ± 3°C for 3 h. Dried samples from individual fish were analysed with one analytical replicate per fish. Pooled samples from milk and dairy products and eggs were analysed with two analytical replicates per sample. Limit of quantification (LOQ) is 0.32 μg/L, or 0.04 mg/kg dry weight. Limit of detection (LOD) is 0.01 μg/L. The measurement uncertainty differs depending on the concentration range and is set to 40% for concentrations between LOQ and 10 × LOQ, and 15% for concentrations >10 × LOQ. The measurement range lies between 0.04 and 90 mg/kg dry weight. The measurement uncertainty is based on the control card of the method along with participation in proficiency tests. ICP-MS is commonly used for the quantitative determination of iodine in biological samples due to its high sensitivity and selectivity (7). Although there are several methods to determine iodine, the sensitivity for iodine in ICP-MS is superior compared to other techniques (21). The freeze-drying method and the ICP-MS method used in this study are accredited according to ISO 17025. Measurement uncertainty is based on internal reproducibility, taken from the control chart of the method along with results from participation in proficiency testing. The method is robust when performed according to the method description. The results obtained from determining iodine content in standard reference materials are listed in Table 3.

**Table 3:** Iodine content in standard reference materials compared with the analysed value and the measured value over time

Reference material	Analysed mean value	Certified value	Measured mean value	RSD %
Skimmed milk powder (ERM-BD 150)	1.50 ± 0.09 mg/kg (*n* = 12)	1.73 ± 0.14 mg/kg	NA	NA
Fish Muscle (ERM-BB 422)	1.23 ± 0.05 mg/kg (*n* = 12)	1.4 ± 0.4 mg/kg	1.26 ± 0.20 mg/kg (*n* = 209)	10

All values are mean values ± standard deviation (SD). Skimmed milk was used for analysing dairy products and fish muscle for analysing fish and egg.

NA, not analysed; RSD, relative standard deviation.

### Statistical analyses

Iodine concentrations in fish of each species sampled in different areas and during different months of the year were compared using one-way ANOVA followed by Tukey’s HSD multiple comparison test. Because of heteroscedasticity, iodine concentrations were log transformed prior to analysis. The relationships between iodine concentration and fish length and condition (*K*-factor = 100 × weight/length^3^), respectively, were examined for each species using Pearson’s linear correlation analysis. Statistical analyses were performed using Statistica 64, version 13.

## Results and discussion

### Iodine content of fish

**Fig. 2:**
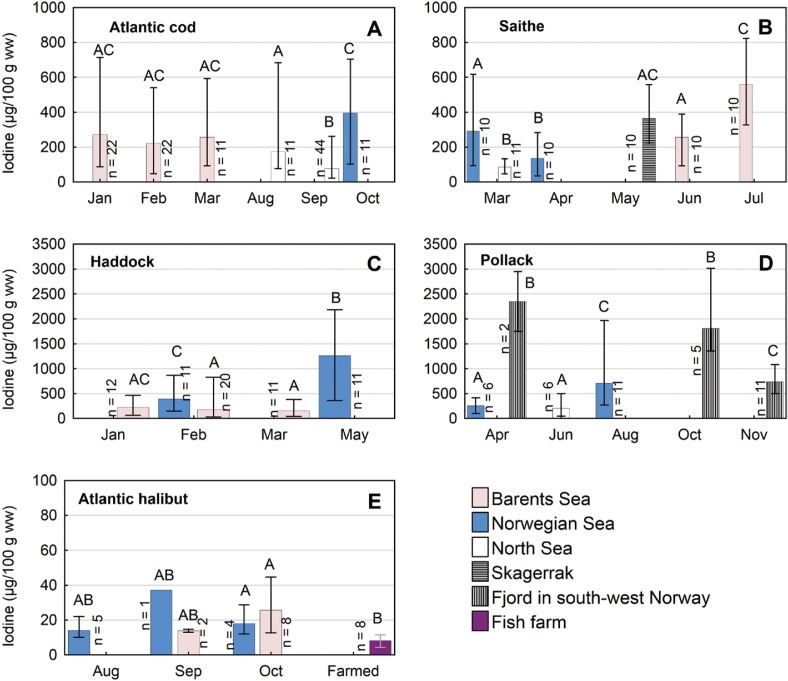
Concentrations of iodine (μg/100 g ww) in A) Atlantic cod (*Gadus morhua*), B) saithe, (*Pollachius virens*), C) haddock (*Melanogrammus aeglefinus*), D) pollack (*Pollachius pollachius*) and E) Atlantic halibut (*Hippoglossus hippoglossus*) sampled during different months in different areas. Mean, minimum and maximum values are given. Significant differences between groups on log_10_-transformed concentrations (one-way ANOVA followed by Tukey HSD) are indicated with different letters. Notice different scaling on the y-axis for the different species.

The average iodine content in fish fillet varied from 21 μg/100 g wet weight (ww) in Atlantic halibut to 790 μg/100 g ww in pollack (Table 4). There was a large variation between individuals within the same species, and between fish of the same species from different geographical areas and/or sampling months (Figure 2). From this dataset, we could not detect any consistency between fish species with regard to which areas or months showed the highest iodine contents. Because this research question was not taken into account during the planning of the sampling and because the main fishery in different areas take place during different times of the year, many of the samples were taken during different months of the year in the different areas. In several cases this precluded the analysis of whether observed differences were geographical or seasonal. For example, Atlantic cod from the North Sea appeared to have lower iodine concentrations than cod from the Barents Sea or the Norwegian Sea. However, most of the samples from the North Sea were taken in September, when no cod were caught in the other two areas (Figure 2A). These cod, caught in September, were relatively large and in good condition (Supplementary Table 1), and since both length and *K*-factor were negatively correlated with iodine content of cod (*r* = −0.38 and −0.35, *p* < 0.001; Supplementary Figures 1 and 2), this may be a possible explanation for the low levels of iodine in cod from the North Sea, particularly in September. In saithe and haddock, iodine concentrations appeared to be higher later in the year, from May on, compared to earlier in the year (Figure 2B,C). For haddock, this could be an effect of spawning, as haddock spawn during April–May (22). If reserves are depleted during spawning to the extent that the total muscle mass is reduced, this could lead to a relative up-concentration of elements such as iodine. The condition (*K*-factor) of the haddock with the highest iodine concentrations, sampled in the Norwegian Sea in May, was indeed reduced (0.96) relative to the haddock sampled in Feburary (1.37; Supplementary Table 1). Saithe spawn earlier in the year, during February–March (22), and the relatively lower concentrations in April compared to later in the year are thus probably not due to spawning. However, iodine is at least partly accumulated from the diet (7), and perhaps increasing iodine content through spring and summer may be caused by enhanced uptake through the feeding season. The condition of the saithe increased slightly as the iodine concentrations increased from March through to July, and in saithe there was a positive correlation between iodine content and *K*-factor (*r* = 0.53, *p* < 0.001; Supplementary Figure 2). In pollack, iodine concentrations were significantly higher in a fjord in southwest Norway both in April and October as compared to all other months in all other areas (Figure 2). These were by far the highest iodine concentrations overall in the study, with mean concentrations of 2,350 and 1,800 μg/100 g in April and October, respectively. Both pollack sampled in April and October were from the same fjord, but the number of fish were low, only seven fish. Pollack from the Norwegian Sea had significantly lower iodine concentrations in April than in August. Condition (*K*-factor) for the pollack from April was, however, high (Supplementary Table 1). This may have been mature fish with large gonads, since pollack spawn during March and April. The high concentrations of iodine in fjords compared to the open sea has limited value for nutritional calculations, as the catch volume of fish from the open sea is much higher than that from the fjords. Fish absorb iodine both from the seawater and from their diet (7). Differences in preferred or available prey for different species and for fish of the same species from different areas may contribute to the large variation in iodine content. The iodine content in Atlantic halibut was low both in the Barents Sea and in the Norwegian Sea, and in both areas the iodine content was much lower than in the four lean codfish investigated. This may be due to a higher fat content in Atlantic halibut than in the four other fish species, since earlier reports have shown that fatty fish generally contain less iodine than lean fish (6). Even though it was the leaner part of the Atlantic halibut muscle (B-cut) (20) that was analysed in this study, the fat content in this part of halibut muscle typically lies between 3 and 5 g per 100 g muscle tissue (23). This is considerably higher than the fat content of the four other species, which typically contain about 0.8–1.5 g fat per 100 g muscle tissue (23). The reason why fatty fish contain lower concentrations of iodine is unknown. The iodine content in muscle of farmed Atlantic halibut was even lower than in wild Atlantic halibut, with an average of 7.8 μg/100 g and a range between 4.4 and 11 μg/100 g for the eight pooled samples (Figure 2). The difference may be due to a higher fat content in muscle of farmed halibut compared to wild halibut, but may also be caused by a low iodine content or bioavailability in the fish feed used for Atlantic halibut in fish farms. For salmon, it has been shown that fish muscle is responsive to iodine supplementations in feed to a certain degree (24). Furthermore, there was a general trend of declining concentrations of iodine in fish feed in Norway during 2000–2006, probably due to reduced use of fish meal in feed production (25). However, there must be other biochemical mechanisms explaining the rather large differences in iodine content between codfish and halibut.

**Table 4:** Iodine content of five different fish species sampled in different geographical areas

Fish species	Catch area	*n*	Mean ± SD μg/100 g	Min.–max. μg/100 g	Mean weight kg	Mean length cm	Food Composition Table^a^ μg/100 g	Sjomatdata^b^
*Atlantic cod*	*All areas*	*121*	*190 ± 160*	*22–720*	*2.8*	*64*	*119^c^*	*93*
Atlantic cod	Barents Sea	55	250 ± 140	47–720	1.8	59		
Atlantic cod	Norwegian Sea	11	400 ± 190	100–700	3.3	69		
Atlantic cod	North Sea	55	96 ± 100	22–680	3.6	68		
*Saithe*	*All areas*	*61*	*280 ± 190*	*35–820*	*1.7*	*53*	*93^d^*	*23*
Saithe	Barents Sea	20	410 ± 200	92–820	1.4	48		
Saithe	Norwegian Sea	20	210 ± 150	35–620	3.0	67		
Saithe	North Sea and Skagerrak	21	220 ± 170	46–560	0.8	43		
*Haddock*	*All areas*	*65*	*400 ± 500*	*35–2,200*	*1.4*	*50*	*320^e^*	*n.a.*
Haddock	Barents Sea	43	180 ± 160	35–830	1.1	47		
Haddock	Norwegian Sea	22	830 ± 650	150–2,200	2.1	56		
*Pollack*	*All areas*	*41*	*790 ± 690*	*48–3,000*	*2.1*	*59*	*143^f^*	*70*
Pollack	Norwegian Sea	17	550 ± 430	100–2,000	2.3	56		
Pollack	North Sea	6	210 ± 160	48–500	2.7	67		
Pollack	Fjords in western Norway	18	1,210 ± 770	497–3,000	1.7	59		
*Atlantic halibut*	*All areas*	*20*	*21 ± 11*	*10–45*	*21*	*115*	*10^g^*	*n.a.*
Atlantic halibut	Barents Sea	10	23 ± 11	13–45	24	122		
Atlantic halibut	Norwegian Sea	10	18 ± 9	10–37	17	109		

Mean ± SD and range (minimum-maximum) are given. Catch area, number of fish (n), mean weight and length of the fish within each area and Food Composition Table (Norwegian Food Safety Authority 2016).

a Norwegian Food Composition Table (30.05.2017), www.matvaretabellen.no

b https://sjomatdata.nifes.no/#search/ (14.03.2018)

c Cod, wild, raw; analysed value from www.sjomatdata.no

d Saithe, raw; analysed value from www.sjomatdata.no (12.07.2010)

e Haddock, raw; Public Health England og Institute of food research (2015). McCance and Widdowson’s The Composition of Foods, Seventh summary edition. Cambridge: The Royal Society of Chemistry. Literature source.

f Pollack, raw; French Agency for Food, Environmental and Occupational Health safety. French Food Composition Table – Ciqual 2012.

g Halibut, Atlantic, raw; Mattilsynet og Sosial- og helsedirektoratet. Analyseprosjekt 2006–2009. Div. fiskeslag. Publisert rapport (2012); ‘Nutritional composition of selected wild and farmed raw fish’.

n.a. – not available.

Table 5 lists iodine contents of 10 canned tuna products from seven different producers, and mean concentrations of the different products varied from 2 to 10 μg/100 g. The concentrations we measured in this study are lower than those of similar products listed with the following concentrations in the Norwegian Food Composition Table: 26 μg/100 g (in water, drained), 10 μg/100 g (in jelly), 26 μg/100 g (in oil, drained) and 10 μg/100 g (in oil, not drained) (26). In addition, the iodine content in tuna was generally lower than in the other fish species in this study (Table 4). Canned tuna may contain different species of tuna, which is one possible explanation for the variation. Unfortunately, the products analysed in this study were not declared with species, catch area or fish size, factors that may affect iodine content.

**Table 5:** Iodine content (mean ± SD) of different types of canned tuna (*n* = 3) purchased in food shops in Bergen, Norway

Type of tuna product (product brand)	Mean ± SD μg/100 g wet weight	Food Composition Table^a^ μg/100 g	Sjomatdata^b^
In water (Rema 1000)	9 ± 3	26^c^	9
In water (Coop)	8 ± 1.5	26^c^	9
In water (Eldorado)	8 ± 1	26^c^	9
In water (First Price)	10 ± 1	26^c^	9
In water (Luxus)	2 ± 1	26^c^	9
In jelly (Rema 1000)	8 ± 4	10^d^	8
In oil (Coop)	7 ± 1	25^c^	8
In oil (Eldorado)	8 ± 2	25^c^	8
In olive oil (Ortiz)	10 ± 3	n.a.	10
In vegetable oil (Ramirez)	8 ± 1	25^c^	8

Mean based on three items from different batch numbers.

a Norwegian Food Composition Table (30.05.2017), www.matvaretabellen.no.

b https://sjomatdata.nifes.no/#search/ (14.03.2018).

c Livsmedelsverket. Livsmedelsdatabas, versjon 2013.01.10. www.slv.se.

d Statens råd for ernæring og fysisk aktivitet og Statens næringsmiddeltilsyn. Analyseprosjekt 2000. Div. matvarer. Internt notat.

n.a. – not available.

### Iodine content of milk and cream milk

Table 6 summarises the results for cow milk and cream milk from the present study, showing the seasonal variation of iodine content in the different products. The table also lists iodine concentrations from the Norwegian Food Composition Table.

**Table 6:** Comparison of iodine content (μg/100 g wet weight) of cow milk and cream milk including seasonal variation and producer, compared with declared values and values listed in the Norwegian Food Composition Table

Dairy product (product brand)	Mean^a^ ± SD	Mean^a^ ± SD	Mean^a^ ± SD	Mean	Mean
	*September–October 2015^b^*	*January–February 2016^b^*	*June–August 2016^b^*	*Declaration from producer*	*Norwegian Food Composition Table^c^*
Low-fat milk 1.2% fat (TINE)	11.7 ± 0.6	16 ± 1	13 ± 1	20	20^d^
Skimmed milk 0.7% fat (TINE)	13 ± 2	16 ± 1	14 ± 2	20	20^d^
Organic low-fat milk 1.2% fat (TINE)	16 ± 6	20 ± 2	15 ± 2	20	20^d^
Organic low-fat 1.2% fat (Røros Meieri)	NA	NA	17^e^	-	20^d^
Low-fat milk 1.0% fat (Q)	12.3 ± 0.6	18 ± 2	12.7 ± 0.6	16	16^d^
Skimmed milk 0.7% fat (Q)	12 ± 0	19 ± 1	13 ± 0	16	20^d^
	*September–October 2015^b^*	*January–February 2016^b^*	*June–July 2016^b^*		
Cream milk 38% (TINE)	7.53 ± 0.06	10.3 ± 0.6	9 ± 1	-	12^d^
	*September–October 2015^f^*	*December 2015–March 2016^f^*	*May–June 2016^f^*		
Food Cream milk 20% fat (TINE)	11 ± 2	12 ± 0	11 ± 0	-	16^d^

a Mean based on three pooled samples

b Due to short durability, the months in each column illustrates both the production, ‘best before’ and shopping month for all milks and the cream milk.

c Norwegian Food Composition Table (30.05.2017), www.matvaretabellen.no

- not declared

NA Not Analysed

d Data from the food industry, analysed values.

e Mean from one pooled sample, hence no SD.

f Due to long durability in food cream milk, the months illustrated are based on the production month.

b/f Due to smaller production lines of both types of cream milks, the months of sampling and analysis (Table 6) were not always the same as for cow milk.

In this study, mean iodine concentrations of summer milk and fall milk were lower than in winter milk, with values between 12 and 17 μg/100 g in summer and fall milk and between 16 and 20 μg/100 g in winter milk, respectively (Table 6). This difference may be explained by a longer period of outdoor pasture feeding and/or differences in the access to iodine fortified cow feed (27). Previous studies have shown seasonal variation of the iodine content in milk with significantly higher iodine concentration in winter milk compared to spring milk or summer milk (27, 28). Due to short durability, the months in each column illustrates both the production, ‘best before’ and shopping month for all milks and the cream milk. Due to long durability in food cream milk, the months illustrated are based on the production month. Due to smaller production lines of both types of cream milks, the months of sampling and analysis (Table 6) were not always the same as for cow milk.

### Iodine content of organic milk

In this study, the iodine concentration of the organic milk was equal to or somewhat higher than the conventional milks within the same seasons (Table 6). This result is in contrast to earlier studies from the United Kingdom (29). However, since we have analysed pooled samples, the number of items are too low and statistical analysis is not applicable. Different practices in organic and conventional farms include routine use, or no use, of vitamins and minerals and the use of fresh foods, among other things, which may be restricted in organic farms. Hence, deficiencies in some minerals can occur in organic farms (29). Rasmussen et al. (30) suggest several explanations for the lower iodine content of their organic milk, less use of iodine-containing mineral mixtures being one of them. In the present study, higher use of such mixtures might be a reason for higher concentrations of iodine in organic milk.

### Iodine content of other dairy products

The concentration of iodine in Norwegian dairy products are summarised in Table 7. The iodine content were compared to the declaration and to the concentratrion listed in the Norwegian Food Composition Table (26).

**Table 7:** Comparison of iodine content (μg/100 g wet weight) from the present study compared with the product declaration and the Norwegian Food Composition Table

Dairy product	Mean^a^ (μg/100 g wet weight)	Declaration from producer	Food Composition Table^b^
*Cow milk with flavour and alternative milk*			
Chocolate-flavoured low-fat cow milk, Q	17	-	18^c^
Soy milk with natural flavour, Alpro	<2	-	1^d^
*Probiotic milk with LGG*			
‘Biola’ with blueberry flavour, TINE	14	16	16^c^
*Yoghurt*			
‘Go’morgen’ with flavour, TINE	13	-	14^e^
Yoghurt with natural flavour, TINE	18	-	13^e^
*White-coloured solid cheese*			
‘Norvegia’, TINE	14	31	31^c^
‘Jarlsberg’, TINE	14	32	37^c^
‘Norsk gulost’, Synnoeve Finden	19	-	-
*White soft and cream cheeses*			
* *Brie, Arla Foods	13	-	43^c^
* *Camembert, TINE	18	45	45^c^
Soft, cream cheese, ‘Snøfrisk’, TINE	46	49	49^c^
Soft, cream cheese, ‘Philadelphia’, Mondalez	14	-	7^f^
*Whey cheese*			
‘Gudbrandsdalsost’, TINE	140	166	166^c^
‘Fløtemysost’, TINE	100	135	135^c^
‘Ekte geitost’, TINE	450	307	306.6^c^,^g^
*Other dairy products*			
Crème fraiche, TINE	10	-	12^c^
Cottage cheese, TINE	15	-	14^h^
Curd with natural flavour, TINE	16	-	20^c^
Sour cream, TINE	9	-	12^c^

a Mean based on two replicates per pooled sample (one pooled sample consists of three items of three different batch numbers)

b Norwegian Food Composition Table (30.05.2017), www.matvaretabellen.no

c Data from the food industry.

d Public Health England og Institute of food research (2015). McCance and Widdowson`s The Composition of Foods, Seventh summary edition. Cambridge: The Royal Society of Chemistry.

e The value is calculated from similar foods.

f Livsmedelsverket. Livsmedelsdatabas, versjon 2016.02.17.

g Goat cheese, whey

LGG, *Lactobacillus rhamnosus GG*

- no declaration

h Danmarks Fødevareforskning. Fødevaredatabanken, versjon 7.01 (2009).

The highest iodine concentrations in the dairy products were found in whey cheese, with concentrations from 100 to 450 μg/100 g (Table 7). In cheese manufacturing, the iodine from milk follows into the whey and not into the curd (9) which is an explaining factor as to why the brown-coloured whey cheese is a rich source of iodine. This type of brown whey cheese is typical in the Norwegian diet. The results from this study show that brown coloured whey cheese is a good source of iodine, which is in accordance with the Norwegian Food Composition Table (26) and a similar study from Norway (27). The presence of goat milk in some of the whey cheeses may also have a positive influence on the concentrations of iodine. This is illustrated by the relatively higher concentrations in both the white-coloured cheese *Snøfrisk* and the brown-coloured whey cheese *Ekte geitost* – both made of goat milk. The latter had more than four times higher concentrations of iodine than the brown-coloured cheese *Fløtemysost*, which is prepared from cow milk. In addition, *Gudbrandsdalsost* contains some goat milk, which may be the reason why this cheese has a higher concentration of iodine than *Fløtemysost*. In accordance with previous analysis of cheese (6), the iodine content did not vary with fat content of the cheese (fat content listed in Table 1). Commonly, cow milk and cream milks are used as a base in the production of other dairy products, which is the reason for analysing these products in only one season. Any seasonal differences found in cow milk and cream milk are assumed to be reflected in dairy products. It is important to have updated data on iodine content of these foods because of the great variation between products (31).

### Analysed concentrations versus declared concentrations of iodine content

The mandatory nutrition declaration of foods in Norway does not include iodine. A large deviation range is accepted by the food safety authorities regarding declaration of minerals. Thus, we cannot conclude that the new analysis is different from the iodine values declared on the products. To minimise seasonal variations, the iodine intake of cows could be controlled, as discussed by Troan et al. (8). Regarding the declared concentrations, one of the producers in this study have based their declaration on their latest analysis of milk from summer and winter 2012. Another producer in this study has based their latest declaration on analysis from 2012 to 2013 (8). Respectively, the first producer has two dairies (one based in south western Norway and one based in eastern Norway), while the latter producer has dairies placed across the country.

### Analysed concentrations versus Norwegian Food Composition Table iodine content

In this study, the mean concentrations of iodine in wild fish were higher than those in the Food Composition Table (Table 4). Thus, estimated intake of iodine from lean fish may be underestimated in dietary surveys from Norway. The range and standard deviations in this study were quite wide. Since concentrations in the Food Composition Table are mean concentrations, analysis of a representative number of samples is necessary in order to estimate intake with high quality. The results for other dairy products than milk in this study were mostly lower than the Food Composition Table (Table 7). In this study, dairy products were sampled and analysed only in autumn, which might be one factor explaining the lower values. In accordance with earlier findings, the iodine varies with seasons, however the sessonal differences are less clear in the present study compared with earlier findings. To reflect the true variation, the Food Composition Table information of nutrient concentrations should include range values.

### Iodine content of hen’s eggs

Table 8 lists updated concentrations of iodine of different types of egg compared to the Food Composition Table. Concentrations measured in whole eggs in this study varied from 23 to 43 μg/100 g, and the values given in the Food Composition Table were within this range, with 35 and 38.5 μg/100 g. Whole eggs from one of the producers from 2016 had lower iodine concentrations than those from the same producer in 2017. This is interesting and may be due to natural variation or changes in ingredients of the hen’s feed. The latter is unfortunately unknown to the authors as this topic is outside the scope of this manuscript. None of the producers have declared iodine contents. Iodine concentrations of eggs in this study were lower as compared to the results from a previous study in Norway where mean concentrations were 45 μg/100 g (range 39–52 μg/100 g, *n* = 90) (6). Regarding iodine, a larger portion is contained in yolk (32). This was also shown in our results, where yolk had iodine concentrations of 57 and 78 μg/100 g and egg white only 2.4 and 3.0 μg/100 g (Table 8). In this study, separate analysis of egg yolk and egg white was only carried out for conventional eggs, while whole egg was analysed for both conventional and organic eggs.

**Table 8:** Comparison of mean iodine content (μg/100 g) in different conventional and organic hen’s eggs

	New analysis			Food Composition Table^a^		
Type of hen’s egg (producer)	Whole egg	Egg white	Yolk	Whole egg	Egg white	Yolk
Conventional (Prior), 2016^b^	23	2.4	57^b^	35^c^	3.4^c^	80.2^c^
Conventional (Prior), 2017^d^	43	NA	NA			
Conventional (Den stolte hane), 2016^b^	33	3	78			
Conventional (Den stolte hane), 2017^d^	41	NA	NA			
Organic (Prior), 2016^b^	43	NA	NA	38.5^c^		
Organic (Prior), 2017^d^	40	NA	NA			
Organic (Den stolte hane), 2016^b^	40	NA	NA			
Organic (Den stolte hane), 2017^d^	31	NA	NA			

a Norwegian Food Composition Table (30.05.2017), www.matvaretabellen.no

b The sample consists of 36 eggs.

c Calculated mean from the Analyses project 2016–2017. Analysis of eggs and chicken (2017): The Norwegian Food Safety Authorities.

d Each sample consists of 18 eggs (*n* = 2).

NA not analysed.

## Conclusion

The fish analysed not only showed a large variation in iodine content between different species but also between individuals within the same species and between locations and/or sampling seasons. Despite these differences, applying our new values would influence intake estimates considerably. Regarding dairy products, the results confirm previous data on seasonal variation of iodine content in milk. This study provideds updated data of the iodine concentration in six fish species, 27 selected Norwegian iodine-rich dairy foods and Norwegian hen’s eggs, which will be made available for the public Food Composition Table.

## Supplementary Material

Iodine content of six fish species, Norwegian dairy products and hen’s egg

## References

[1] Abel MH, Caspersen IH, Meltzer HM, Haugen M, Brandlistuen RE, Aase H, et al. Suboptimal maternal iodine intake is associated with impaired child neurodevelopment at 3 years of age in the Norwegian Mother and Child Cohort Study. J Nutr 2017; 147(7): 1314–24. 28515161 10.3945/jn.117.250456

[2] Hynes KL, Otahal P, Hay I, Burgess JR Mild iodine deficiency during pregnancy is associated with reduced educational outcomes in the offspring: 9-year follow-up of the gestational iodine cohort. J Clin Endocrinol Metab 2013; 98(5): 1954–62. 23633204 10.1210/jc.2012-4249

[3] Pearce EN, Lazarus JH, Moreno-Reyes R, Zimmermann MB Consequences of iodine deficiency and excess in pregnant women: an overview of current knowns and unknowns. Am J Clin Nutr 2016; 104(Suppl 3): 918S–23S. 27534632 10.3945/ajcn.115.110429 PMC5004501

[4] Bath SC, Rayman MP Iodine deficiency in the U.K.: an overlooked cause of impaired neurodevelopment? Proc Nutr Soc 2013; 72(2): 226–35. 23570907 10.1017/S0029665113001006

[5] Gunnarsdottir I, Gustavsdottir AG, Thorsdottir I Iodine intake and status in Iceland through a period of 60 years. Food Nutr Res 2009; 53: 1925. 10.3402/fnr.v53i0.1925 PMC2691155 19503752

[6] Dahl L, Johansson L, Julshamn K, Meltzer HM The iodine content of Norwegian foods and diets. Public Health Nutr 2004; 7(4): 569–76. 15153264 10.1079/PHN2003554

[7] Julshamn K, Dahl L, Eckhoff K Determination of iodine in seafood by inductively coupled plasma/mass spectrometry. J AOAC Int 2001; 84(6): 1976–83. 11767171

[8] Troan G, Dahl L, Meltzer HM, Abel MH, Indahl UG, Haug A, et al. A model to secure a stable iodine concentration in milk. Food Nutr Res 2015; 59: 29829. 26689316 10.3402/fnr.v59.29829 PMC4685975

[9] Haldimann M, Alt A, Blanc A, Blondeau K Iodine content of food groups. J Food Compos Anal 2005; 18(6): 461–71.

[10] WHO, UNICEF, ICCID Assessment of iodine deficiencydisorders and monitoring their elimination: a guide for programme managers. Geneva: World Health Organization; 2007.

[11] Nystrom HF, Brantsaeter AL, Erlund I, Gunnarsdottir I, Hulthen L, Laurberg P, et al. Iodine status in the Nordic countries – past and present. Food Nutr Res 2016; 60: 31969. 27283870 10.3402/fnr.v60.31969 PMC4901513

[12] Volzke H, Caron P, Dahl L, de Castro JJ, Erlund I, Gaberscek S, et al. Ensuring effective prevention of iodine deficiency disorders. Thyroid 2016; 26(2): 189–96. 26700864 10.1089/thy.2015.0543

[13] Rasmussen LB, Carle A, Jorgensen T, Knudsen N, Laurberg P, Pedersen IB, et al. Iodine intake before and after mandatory iodization in Denmark: results from the Danish Investigation of Iodine Intake and Thyroid Diseases (DanThyr) study. Br J Nutr 2008; 100(1): 166–73. 18208635 10.1017/S0007114507886387

[14] Julshamn K, Duinker A, Nilsen BM, Frantzen S, Maage A, Valdersnes S, et al. A baseline study of levels of mercury, arsenic, cadmium and lead in Northeast Arctic cod (Gadus morhua) from different parts of the Barents Sea. Mar Pollut Bull 2013; 67(1–2): 187–95. 23260646 10.1016/j.marpolbul.2012.11.038

[15] Julshamn K, Duinker A, Nilsen BM, Nedreaas K, Maage A A baseline study of metals in cod (Gadus morhua) from the North Sea and coastal Norwegian waters, with focus on mercury, arsenic, cadmium and lead. Mar Pollut Bull 2013; 72(1): 264–73. 23706615 10.1016/j.marpolbul.2013.04.018

[16] Frantzen S, Maage A Fremmedstoffer i villfisk med vekt på kyst-nære farvann [Contaminants in wild caught fish with emphasis on coastal waters. Tusk, ling and bycatch species. Results for samples collected during 2013-2015]. Brosme, lange og bifangstarter. 2016 Available from: https://nifes.hi.no/report/rapport-villfisk-2016/

[17] Nilsen BM, Nedreaas K, Maage A Kartlegging av fremmedstoffer i Atlantisk kveite (Hippoglossus hippoglossus). [Baseline study of contaminants in Atlantic halibut (Hippoglossus hippoglossus)]. 2016 Available from: https://nifes.hi.no/report/atlantisk-kveite-sluttrapport/

[18] Nilsen BM, Julshamn K, Duinker A, Nedreaas K, Maage A Basisundersøkelse av fremmedstoffer i sei (Pollachius virens) fra Nordsjøen. [Baseline study of contaminants in saithe (Pollachius virens) from the North Sea]. 2013 Available from: https://nifes.hi.no/report/basisundersokelse-av-fremmedstoffer-i-sei-pollachius-virens-fra-nordsjoen/

[19] Nilsen BM, Julshamn K, Duinker A, Nedreaas K, Maage A Basisundersøkelse av fremmedstoffer i sei (Pollachius virens) fra Norskehavet og Barentshavet. [Baseline study of contaminants in saithe (Pollachius virens) from the Norwegian Sea and the Barents Sea] 2013 Available from: https://nifes.hi.no/report/basisundersokelse-fremmedstoffer-sei-norskehavet-barentshavet/

[20] Nortvedt R, Tuene S Body composition and sensory assessment of three weight groups of Atlantic halibut (Hippoglossus hippoglossus) fed three pellet sizes and three dietary fat levels. Aquaculture 1998; 161(1–4): 295–313.

[21] Shelor CP, Dasgupta PK Review of analytical methods for the quantification of iodine in complex matrices. Anal Chim Acta 2011; 702(1): 16–36. 21819856 10.1016/j.aca.2011.05.039

[22] Olsen E, Aanes S, Mehl S, Holst JC, Aglen A, Gjøsæter H Cod, haddock, saithe, herring, and capelin in the Barents Sea and adjacent waters: a review of the biological value of the area. ICES J Marine Sci 2010; 67(1): 87–101.

[23] IMR (Institute of Marine Research). Seafood data. 2018 Available from: https://sjomatdata.nifes.no/#/substance/402/-1 [cited 19 March 2018].

[24] Julshamn K, Maage A, Waagbø R, Lundebye A A preliminary study on tailoring of fillet iodine concentrations in adult Atlantic salmon (Salmo salar L.) through dietary supplementation. Aquacult Nutr 2006; 12: 45–51.

[25] Sissener NH, Julshamn K, Espe M, Maage A Surveillance of selected nutrients, additives and undesirables in commercial Norwegian fish feeds in the years 2000–2010. Aquacult Nutr 2013; 19(4): 555–572.

[26] Norwegian Food Safety Authority TNDoH, University of Oslo Norwegian food composition database. 2016 Available from: http://matvaretabellen.no/?language=en [cited 23 June 2017].

[27] Dahl L, Opsahl JA, Meltzer HM, Julshamn K Iodine concentration in Norwegian milk and dairy products. Br J Nutr 2003; 90(3): 679–85. 13129475 10.1079/bjn2003921

[28] Cressey PJ Iodine content of New Zealand dairy products. J Food Compos Anal 2003; 16(1): 25–36.

[29] Bath SC, Button S, Rayman MP Iodine concentration of organic and conventional milk: implications for iodine intake. Br J Nutr 2012; 107(7): 935–40. 21781365 10.1017/S0007114511003059

[30] Rasmussen LB, Larsen EH, Ovesen L Iodine content in drinking water and other beverages in Denmark. Eur J Clin Nutr 2000; 54(1): 57–60. 10694773 10.1038/sj.ejcn.1600893

[31] Norwegian Scientific Committee for Food Safety V. Assessment of salt fortified with iodine and flouride. Available from: https://www.vkm.no/download/18.2994e95b15cc5450716d638b/1500307741080/895624d06a.pdf

[32] Travnicek J, Kroupova V, Herzig I, Kursa J Iodine content in consumer hen eggs. Vet Med (Praha) 2006; 51(3): 93.

