# Supplementary material for: Iodine content of six fish species, Norwegian dairy products and hen’s egg
Source: Food Nutr Res. 2018 May 24;62:10.29219/fnr.v62.1291. doi: 10.29219/fnr.v62.1291 (PMC5971469; doi:10.29219/fnr.v62.1291)
Supplement: Iodine content of six fish species, Norwegian dairy products and hen’s egg [file FNR-62-1291-s001.docx]

# Supplementary information

Supplementary Figure 1. Correlation between iodine concentration (given as log_10_I) and fish length (cm) for the different fish species.

Supplementary Figure 2. Correlation between iodine concentration (given as log_10_I) and fish condition (K-factor = 100 x weight/length^3^) for the different fish species.

Supplementary Table 1. Iodine content (µg/100 g ww) of fillet, fish length (l), fish weight (w) and fish condition given as K-factor (100 x w/l^3^) for the different fish species categorized by sea area and month of capture.

|  |  |  |  | I  (µg/100 g) | Length  (cm) | Weight  (g) | K-factor  (100 x w/l^3^) |
| --- | --- | --- | --- | --- | --- | --- | --- |
| Species | Area | Month | N | Mean ± sd | Mean ± sd | Mean ± sd | Mean ± sd |
|  |  |  |  | Min - max | Min - max | Min - max | Min - max |
| Atlantic cod | Barents Sea | Jan | 22 | 272 ± 152 | 61.9 ± 6.9 | 2185 ± 756 | 0.883 ± 0.078 |
|  |  |  |  | 88 - 715 | 51 – 71 | 1075 - 3431 | 0.77 - 1.1 |
|  | Barents Sea | Feb | 22 | 222 ± 141 | 57.0 ± 2.7 | 1599 ± 249 | 0.862 ± 0.075 |
|  |  |  |  | 47 - 541 | 51 – 62 | 1146 - 2184 | 0.73 - 1.0 |
|  | Barents Sea | Mar | 11 | 255 ± 134 | 55.6 ± 3.6 | 1520 ± 254 | 0.878 ± 0.054 |
|  |  |  |  | 92 - 593 | 51 – 60 | 1150 - 1855 | 0.78 - 0.98 |
|  | Norw. Sea | Oct | 11 | 396 ± 188 | 69.5 ± 4.5 | 3292 ± 578 | 0.980 ± 0.119 |
|  |  |  |  | 103 - 705 | 62 – 76 | 2141 - 4275 | 0.83 - 1.2 |
|  | North Sea | Aug | 11 | 175 ± 171 | 62.0 ± 5.1 | 2977 ± 780 | 1.24 ± 0.20 |
|  |  |  |  | 77 - 684 | 51 – 72 | 1874 - 4270 | 0.89 - 1.5 |
|  | North Sea | Sep | 44 | 75.8 ± 60.0 | 69.9 ± 4.0 | 3747 ± 496 | 1.10 ± 0.12 |
|  |  |  |  | 22 - 261 | 62 – 77 | 2862 - 4834 | 0.89 - 1.4 |
| Haddock | Barents Sea | Jan | 12 | 224 ± 132 | 51.8 ± 5.6 | 1588 ± 509 | 1.10 ± 0.13 |
|  |  |  |  | 62 - 465 | 40.5 - 57.5 | 674 - 2154 | 0.89 - 1.3 |
|  | Barents Sea | Feb | 20 | 177 ± 195 | 46.8 ± 4.6 | 1038 ± 311 | 0.98 ± 0.08 |
|  |  |  |  | 35 - 825 | 41.5 - 52.5 | 676 - 1405 | 0.82 - 1.2 |
|  | Barents Sea | Mar | 11 | 152 ± 108 | 42.4 ± 2.0 | 800 ± 148 | 1.04 ± 0.11 |
|  |  |  |  | 41 - 382 | 40.5 - 46.5 | 623 - 1152 | 0.85 - 1.2 |
|  | Norw. Sea | Feb | 11 | 390 ± 252 | 56.0 ± 1.6 | 2400 ± 222 | 1.37 ± 0.13 |
|  |  |  |  | 151 - 866 | 54.5 - 58.5 | 2135 - 2831 | 1.1 - 1.6 |
|  | Norw. Sea | May | 11 | 1260 ± 644 | 56.6 ± 1.0 | 1733 ± 131 | 0.96 ± 0.10 |
|  |  |  |  | 361 - 2186 | 55.5 - 58.0 | 1450 - 1897 | 0.78 - 1.1 |
| Pollock | Norw. Sea | Apr | 6 | 259 ± 108 | 56.7 ± 5.0 | 3744 ± 731 | 2.09 ± 0.45 |
|  |  |  |  | 100 - 416 | 50 – 65 | 2717 - 4885 | 1.2 - 2.4 |
|  | Norw. Sea | Aug | 11 | 708 ± 455 | 55.9 ± 1.5 | 1585 ± 545 | 0.901 ± 0.299 |
|  |  |  |  | 270 - 1965 | 53.5 - 58.0 | 55 - 2088 | 0.03 - 1.2 |
|  | North Sea, w of | Jun | 6 | 208 ± 156 | 66.7 ± 5.1 | 2722 ± 675 | 0.902 ± 0.061 |
|  | Shetland |  |  | 48 - 500 | 60 – 74 | 1736 - 3656 | 0.80 - 0.97 |
|  | Fjord in sw Norway | Apr | 2 | 2349 ± 850 | 53.5 ± 0.7 | 1186 ± 61 | 0.774 ± 0.009 |
|  |  |  |  | 1748 - 2950 | 53 - 54 | 1143 - 1229 | 0.77 - 0.78 |
|  | Fjord in sw Norway | Oct | 5 | 1806 ± 713 | 57.2 ± 2.3 | 1260 ± 77 | 0.678 ± 0.086 |
|  |  |  |  | 1355 - 3015 | 54 – 60 | 1183 - 1381 | 0.59 - 0.79 |
|  | Fjord in sw Norway | Nov | 11 | 738 ± 194 | 60.3 ± 2.7 | 1995 ± 225 | 0.914 ± 0.103 |
|  |  |  |  | 497 - 1082 | 57.0 - 63.0 | 1704 - 2404 | 0.74 - 1.1 |
| Saithe | Barents Sea | Jun | 10 | 256 ± 108 | 56.6 ± 1.3 | 1996 ± 197 | 1.10 ± 0.06 |
|  |  |  |  | 92 - 389 | 54 - 58.5 | 1660 - 2371 | 1.0 - 1.2 |
|  | Barents Sea | Jul | 10 | 561 ± 136 | 40.3 ± 1.3 | 738 ± 61 | 1.13 ± 0.07 |
|  |  |  |  | 326 - 824 | 38 – 42 | 625 - 826 | 0.98 - 1.2 |
|  | Norw. Sea | Mar | 10 | 291 ± 164 | 62.4 ± 4.6 | 2444 ± 580 | 0.990 ± 0.078 |
|  |  |  |  | 93 - 617 | 57 - 68 | 1586 - 3292 | 0.86 - 1.1 |
|  | Norw. Sea | Apr | 10 | 136 ± 80 | 70.7 ± 2.1 | 3627 ± 335 | 1.03 ± 0.10 |
|  |  |  |  | 35 - 283 | 68 – 74 | 3141 - 4305 | 0.89 - 1.2 |
|  | North Sea/ Skagerrak | Mar | 11 | 85.7 ± 26 | 44.3 ± 1.3 | 780 ± 119 | 0.894 ± 0.084 |
|  |  |  |  | 46 - 133 | 42 – 46 | 574 - 909 | 0.72 - 1.0 |
|  | North Sea/ Skagerrak | May | 10 | 365 ± 120 | 42.6 ± 0.5 | 815 ± 62 | 1.05 ± 0.07 |
|  |  |  |  | 218 - 558 | 42 – 43 | 703 - 893 | 0.95 - 1.15 |
| Atlantic halibut | Barents Sea | Sep | 2 | 13.8 ± 1.3 | 119 ± 2 | 21750 ± 3606 | 1.30 ± 0.15 |
|  |  |  |  | 13 - 15 | 117 – 120 | 19200 - 24300 | 1.2 - 1.4 |
|  | Barents Sea | Oct | 8 | 25.6 ± 13.6 | 123 ± 18 | 24434± 9990 | 1.26 ± 0.09 |
|  |  |  |  | 13 - 45 | 94 – 141 | 11100 - 39400 | 1.2 - 1.4 |
|  | Norw. Sea | Aug | 5 | 14.0 ± 4.7 | 109 ± 11 | 16837 ± 5465 | 1.26 ± 0.05 |
|  |  |  |  | 010 - 22 | 98 – 125 | 11180 - 24860 | 1.2 - 1.3 |
|  | Norw. Sea | Sep | 1 | 37 | 100 | 11300 | 1.1 |
|  | Norw. Sea | Oct | 4 | 18.0 ± 7.3 | 111 ± 14 | 19823 ± 10953 | 1.35 ± 0.22 |
|  |  |  |  | 12 - 29 | 99 – 131 | 12460 - 36030 | 1.1 - 1.6 |
|  | Farmed | Farmed | 8 | 7.8 ± 2.3 |  |  |  |
|  |  |  |  | 4.4 - 11 |  |  |  |
